# Steep left ventricle to aortic root angle is independently associated with dynamic left ventricular outflow tract gradient in hypertrophic cardiomyopathy: a novel association using 3-dimensional multi-modality imaging

**DOI:** 10.1186/1532-429X-11-S1-P197

**Published:** 2009-01-28

**Authors:** Deborah H Kwon, Nicholas G Smedira, Zoran B Popovic, Bruce W Lytle, Randolph M Setser, Marn Thamilarasan, Paul Schoenhagen, Scott D Flamm, Harry Lever, Milind Y Desai

**Affiliations:** 1 Cleveland Clinic Foundation, Cleveland, OH USA; 2 Cleveland Clinic, Cleveland, OH USA

**Keywords:** Cardiac Magnetic Resonance, Hypertrophic Cardiomyopathy, Root Angle, Left Ventricular Outflow Tract Gradient, LVOT Obstruction

## Introduction

**Figure 1:**
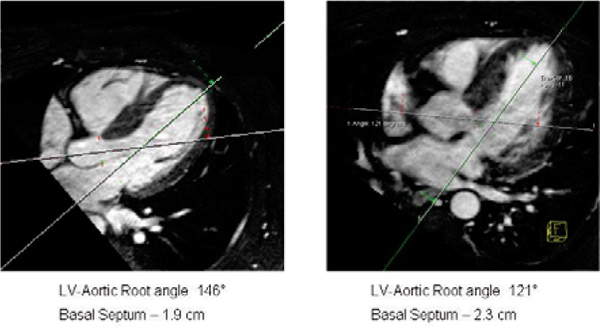
**Quantification of the LVARA in the 5 chamber view**.

Hypertrophic cardiomyopathy (HCM) patients with similar basal septal hypertrophy (BSH) can have significantly different degrees of dynamic obstruction, quantified by the left ventricular outflow tract gradient (LVOTG). Furthermore, an elevated LVOTG can be seen even with minimal BSH. Using advanced 3-dimensional whole-heart sequence on cardiac magnetic resonance (CMR), we observed a spectrum of acuity in the LV to aortic root angle (LVARA) in our study population of HCM patients (Figure 1).

## Purpose

In HCM patients, we sought to determine if a steeper LVARA was associated with an increased LVOTG, independent of BSH.

## Methods

We studied 153 consecutive patients (≤ 65 years) with echo-documented HCM who underwent standard CMR (1.5 T Siemens Avanto, Erlangen, Germany) along with whole-heart 3D MR angiogram which was a navigator-assisted free-breathing, ECG-triggered, fat saturated, T2-prepared, segmented 3D SSFP sequence. Imaging parameters were as follows: TR = 3.8 ms, TE = 1.9 ms, flip angle = 70°, acquired matrix = 175–216 (phase direction) by 256 (readout direction) points (no interpolation), Grappa factor of 2, 24 reference lines and sampling bandwidth = ± 125 kHz. Typically, 60–70 slices were acquired at 1.5 mm thickness (interpolated) in order to cover the heart. The in-plane resolution was typically 1.3–1.6 mm. Images were acquired during a 150 msec data acquisition window in mid-to-late diastole. LVARA, LV volumes (indexed to body surface area) and BSH were measured on CMR. Maximal (resting or provocable) LVOT gradients were recorded on echocardiography. Inter and intra-observer concordance of LV-aortic root angle measurement was assessed in 14 HCM patients using intraclass correlation coefficient (ICC).

## Results

The baseline characteristics were as follows: mean age 46 ± 14 years, 68 % male, 36 % hypertensives, 73 % were on beta-blockers. The mean LVARA, maximal LVOT gradient, BST, end systolic and end diastolic volume indexes were 134° ± 10, 82 mm Hg ± 60, 1.98 cm ± 0.6, 32 ml/m^2^ ± 11 and 84 ml/m^2^ ± 16, respectively. Univariate and multivariate associations between maximal LVOT gradient and various factors are shown in Table 1. Age correlated with LVARA (r = -0.56, p < 0.001). The ICC for intra-observer (0.91) and inter-observer (0.88) concordance of LV-aortic root angles in the HCM group was very high (both p-values < 0.0001).

**Table 1:** Univariated and multivariate regression analysis testing the association between maximal LVOT gradient

	Univariate Analysis Beta	Univariate Analysis p value	Multivariate Analysis Beta	Multivariate Analysis p value
Left ventricle to aortic root angle	-0.34	< 0.001	-0.24	0.01
Age	0.23	0. 01	0.19	0.05
End-systolic volume index	-0.20	0.02	-0.16	0.06
Gender	0.05	0.5		
Hypertension	0.06	0.5		
Atrial fibrillation	-0.05	0.5		
Beta blocker use	0.05	0.5		
End-diastolic volume index	0.04	0.7		
Basal end-diastolic interventricular septal thickness	0.02	0.8		

## Conclusion

In patients with HCM, a steeper LVARA predicts an increased LVOTG, independent of BSH. Steep LVARA, which likely represents accelerated remodelling of the LVOT, may explain why HCM patients with similar degree of BST have different degrees of dynamic LVOT obstruction. The prognostic importance of steep LVARA remains to be determined. This study demonstrates the clinical utility of multi-modality imaging, including CMR, in further understanding the varied phenotypic presentation and complex pathophysiology found in HCM.

